# Oral Bacteriotherapy Reduces the Occurrence of Chronic Fatigue in COVID-19 Patients

**DOI:** 10.3389/fnut.2021.756177

**Published:** 2022-01-12

**Authors:** Letizia Santinelli, Luca Laghi, Giuseppe Pietro Innocenti, Claudia Pinacchio, Paolo Vassalini, Luigi Celani, Alessandro Lazzaro, Cristian Borrazzo, Massimiliano Marazzato, Lorenzo Tarsitani, Alexia E. Koukopoulos, Claudio M. Mastroianni, Gabriella d'Ettorre, Giancarlo Ceccarelli

**Affiliations:** ^1^Department of Public Health and Infectious Diseases, Sapienza University of Rome, Rome, Italy; ^2^Department of Agricultural and Food Sciences, University of Bologna, Bologna, Italy; ^3^Interdepartmental Centre for Agri-Food Industrial Research, University of Bologna, Bologna, Italy; ^4^Department of Human Neurosciences, Policlinico Umberto I, Sapienza University of Rome, Rome, Italy

**Keywords:** chronic fatigue, COVID-19, probiotics, metabolomics, FAS, Arginine, Asparagine, Lactate

## Abstract

Long COVID refers to patients with symptoms as fatigue, “brain fog,” pain, suggesting the chronic involvement of the central nervous system (CNS) in COVID-19. The supplementation with probiotic (OB) would have a positive effect on metabolic homeostasis, negatively impacting the occurrence of symptoms related to the CNS after hospital discharge. On a total of 58 patients hospitalized for COVID-19, 24 (41.4%) received OB during hospitalization (OB+) while 34 (58.6%) taken only the standard treatment (OB–). Serum metabolomic profiling of patients has been performed at both hospital acceptance (T0) and discharge (T1). Six months after discharge, fatigue perceived by participants was assessed by administrating the Fatigue Assessment Scale. 70.7% of participants reported fatigue while 29.3% were negative for such condition. The OB+ group showed a significantly lower proportion of subjects reporting fatigue than the OB– one (*p* < 0.01). Furthermore, OB+ subjects were characterized by significantly increased concentrations of serum Arginine, Asparagine, Lactate opposite to lower levels of 3-Hydroxyisobutirate than those not treated with probiotics. Our results strongly suggest that in COVID-19, the administration of probiotics during hospitalization may prevent the development of chronic fatigue by impacting key metabolites involved in the utilization of glucose as well as in energy pathways.

## Introduction

Since its inception, COVID-19 immediately proved to be an aggressive respiratory syndrome capable of degenerating into pneumonia, ARDS (Acute Respiratory Distress Syndrome), multiorgan failure, and death. Eventually, the massive rise of new cases over time, highlighted the presence of post-SARS-CoV-2-chronic symptoms following the resolution of the disease's critical and infective phase. A distinctive characteristic of the “Long COVID” (1) is post-exertion malaise, worsening symptoms following physical or mental exertion occurring within 12–48 h of the effort and requiring an extended recovery period (2–5). The clinical picture of the Long COVID patients reporting fatigue is similar to what was observed in individuals with myalgic encephalopathy/chronic fatigue syndrome (ME/CFS), e.g., several months of profound exhaustion, abysmal performance, and short-term memory problems.

All the current hypotheses, e.g., mitochondrial dysfunction, immune system dysregulation, nitric oxide dysmetabolism, hypothalamic–pituitary–adrenal disruption, and genetic predisposition, call into question the microbes' role in the intestines. Abdominal discomfort, nausea, diarrhea, and vomiting are clinically evident in about 50% of COVID-19 patients (6). These medical signs and symptoms are more frequent in the virus infected individuals with an altered microbiome due to pre-existing conditions such as chronic diseases, inflammation, drug treatments, and age (7). Levels of bacteria with probiotic properties [e.g., *Lactobacillus and Bifidobacterium*; (8)] and other beneficial symbionts are lower in hospitalized COVID-19 patients. By contrast, opportunistic pathogens (e.g., Streptococcus, Rothia, and Actinomyces) are higher (9, 10). Notably, these changes may persist after respiratory symptoms resolution and usually correlate with COVID-19 severity (10).

We have already demonstrated and published the benefits of oral bacteriotherapy for COVID-19 patients during the hospital stay *quo ad vitam* (11–15). In this paper, we report the efficacy of our therapeutic approach *quo ad valetudinem*.

## Materials and Methods

### Participants

We conducted a retrospective observational study at the Division of Infectious Diseases, Department of Public Health and Infectious Diseases, Umberto I Hospital of Sapienza University of Rome (Italy), including 58 patients hospitalized from March the 1st and April the 30th 2020 with confirmed COVID-19 and discharged to home care.

All the hospitalized patients received therapeutic regimens including one or more of the following antimicrobial agents: hydroxychloroquine (200 mg twice a day for 7 days), azithromycin (500 mg once a day for 5 days), antiviral therapy including lopinavir–ritonavir (400/100 mg twice a day), or darunavir–cobicistat (800/150 mg once a day) for 14 days. Low molecular weight heparin was administered for prevention of deep vein thrombosis, as recommended at the time by the Italian Society of Infectious Diseases (16). Tocilizumab (8 mg/kg up to a maximum of 800 mg per dose with an interval of 12 h for two times) was administered in case of high serum IL-6 or of significant worsening of the respiratory picture in case of unavailability of IL-6 dosage. The data source for patient information analysis was derived from electronic medical records in the Hospital Electronic Information System. The variables considered for the study included: (1) age, gender, admission, and discharge date from the hospital, length of hospitalization, (2) cardiovascular (CV) disease. We used the Charlson score to predict the 1-year mortality for a patient with a range of comorbid conditions (17).

Among all participants, a group of 24 subjects received oral bacteriotherapy (OB) during the entire hospitalization period (T1) [median: 23 days (IQR: 19–38 days)] (OB+). Another group of 34 individuals (OB–) was not supplement with oral bacteriotherapy [median hospitalization period: 21 days (IQR: 18–26 days)]. Patients admitted to the ward received supplementation with oral bacteriotherapy, in addition to the best available therapy, in case of intestinal symptoms (11). Known or suspected allergy or intolerance to oral bacteriotherapy formulation was considered a contraindication to the prescription of supplementation.

The commercial oral bacteriotherapy formulation (SLAB51; currently sold under the brand Sivomixx800®, Ormendes, Switzerland) was composed of *Streptococcus thermophilus* DSM 32245®, *Bifidobacterium lactis* DSM 32246®, *Bifidobacterium lactis* DSM 32247®, *Lactobacillus acidophilus* DSM 32241®, *Lactobacillus helveticus* DSM 32242®, *Lactobacillus paracasei* DSM 32243®, *Lactobacillus plantarum* DSM 32244®, and *Lactobacillus brevis* DSM 27961®. The formulation was administered in three equal doses per day for a total of 2,400 billion bacteria per day.

We applied the following exclusion criteria: clinically evident cognitive impairment and inadequate knowledge of the Italian language, history of or current diseases of the small or large intestine, any current or previous systemic malignancy, and pregnancy. Nasopharyngeal swabs and blood samples were collected at hospital admission (T0) and before discharge (T1) from all SARS-CoV-2-positive patients. Six months after discharge, all patients were also contacted by telephone by trained clinical raters, and Fatigue Assessment Scale (FAS) questionnaire was administered.

The Ethical Committee of the Sapienza University of Rome approved the study (num. Rif. 109/2020). All study participants gave written informed consent.

### RT-qPCR Detection of SARS-CoV-2 RNA

Viral RNA was extracted from nasopharyngeal swabs using Versant SP 1.0 Kit (Siemens Healthcare Diagnostics), as previously described (18). Briefly, 10 μl of extracted RNA was reverse-transcribed and simultaneously amplified by a real-time RT-PCR system (RealStar SARS-CoV-2 RT-PCR, Altona Diagnostics), targeting E and S viral genes.

### Fatigue Assessment Scale (FAS)

Six months after hospital discharge, participants were contacted by telephone by trained clinical raters, and completed the Fatigue Assessment Scale test, a 10 items questionnaire used to assess perceived fatigue (19). FAS is validated for patient affected by rheumatological disease (20), sarcoidosis, (21) but also for general population (20). It has been already used in COVID-19 patients to evaluate fatigue in post-COVID setting (22, 23). Of the 10 questions, 5 assess physical fatigue (questions 1–2, 4–5, and 10) and 5 mental fatigue (questions 3 and 6–9). Responses are on a 5-point Likert scale (from 1 = never to 5 = always). The FAS total score ranges from 10 to 50, with higher score indicating more fatigue. Total scores below 22 indicate “non-fatigued” persons, scores higher or equal to 22 indicate “fatigued” patients, and scores higher or equal to 35 indicate extreme fatigue.

### Serum Metabolome Analysis

For metabolomics investigation by Proton Nuclear Magnetic Resonance (^1^H-NMR), a standard solution was prepared with 3-(trimethylsilyl)-propionic-2,2,3,3-d4 acid (TSP) sodium salt, 10 mM, and sodium azide, 2 mM, in D_2_O, set at pH 7.00 with a 1-M phosphate buffer.

Whole blood was left to stand for 30′ at 20°C before being centrifuged at 3,000 rpm for 15′ at 4°C for serum isolation, within 2 h after withdrawal. The samples were then stored at −80°C prior to investigation. Each thawed aliquot was centrifuged at 18,630 g for 10 min at 4°C degrees; 0.7 mL of supernatant were added to 0.1 mL of the standard solution and centrifuge again. ^1^H-NMR spectra were recorded at 298 K with an AVANCE III spectrometer (Bruker, Milan, Italy) operating at a frequency of 600.13 MHz. The signal due to the residual hydrogen deuterium oxide was suppressed by presaturation, whereas broad signals from slowly tumbling molecules were removed by including a Carr–Purcell–Meiboom–Gill filter (24) to a free induction decay sequence. The filter was made up by a train of 400 echoes separated by 800 μs, for a total time of 328 ms. Each spectrum was acquired by summing up 256 transients using 32 K data points over a 7211.54 Hz spectra (for an acquisition time of 2.27 s). The recycle delay was set to 8 s, considering the longitudinal relaxation time of the protons under investigation. Spectra were adjusted for phase and baseline in Topspin ver. 3.5 (Bruker, Milan, Italy).

Signals were assigned by comparing their chemical shift and multiplicity with the Human Metabolome Database (25) and Chenomx software library (Chenomx Inc., Edmonton, Canada, ver. 10). Any other further processing was performed in R computational language (www.r-project.org). Moreover, molecules' quantification was performed in the first sample acquired by employing an external standard, while the spectra from the other samples were adjusted toward the first by probabilistic quotient normalization (26). Integration of the signals was performed for each molecule by means of rectangular integration.

### Statistical Analysis

The statistical analyses were performed using GraphPad Prism software, version 5.0 (GraphPad Software Inc., La Jolla, California, USA) and Statistical package for social science (SPSS software), version 22 (IBM SPSS, Chicago, III). The continuous data were presented as means with Standard Deviation (±SD), and medians with Interquartile Range (IQR: 25–75%), and the presence of statistically significant differences between groups were assessed by the Student's *t*-test or Mann–Whitney *U*-test. The dichotomous variables were described as simple frequencies (*n*) and percentages (%) and then compared by the Fisher's exact test or χ^2^-test for the two groups.

Metabolic concentration differences between SARS-CoV-2-infected patients receiving or not oral bacteriotherapy formulation were analyzed using the Mann–Whitney *U*-test. The Wilcoxon signed-rank test for paired samples was used to evaluate Metabolic concentration differences between T0 and T7 in OB+ and in OB– patients. A point-biserial Pearson's correlation was calculated to assess the correlation between oral bacteriotherapy supplementation and serum metabolites concentration. A *p* < 0.05 was considered statistically significant.

## Results

### Study Population

A total of 58 SARS-CoV-2-infected patients were enrolled in the study. Demographic and clinical characteristics of the whole population at the hospital admission (T0) are reported in Table 1. During hospitalization, 79.3% (46/58) of the patients received supplemental oxygen therapy, 88% (51/58) of cases were treated with hydroxychloroquine, 31% (18/58) with azithromycin, 48.3% (28/58) with anti-IL-6 agent (Tocilizumab) and 29.3% (17/58) were treated with antiviral therapy.

**Table 1:** Demographic and clinical characteristic of study population at T0.

**Parameters**	**Median (IQR 25–75%)**	***n* (%)**
Gender. Male	–	37 (64)
Age (years)	63 (56–70)	–
White blood cells (mmc)	5,625 (4072.5–7,105)	
Lymphocytes (mmc)	805 (640–1,200)	–
Lymphocytes (%)	16 (10.5–23.43)	–
Glucose (mg/dL)	102 (88.25–128.5)	
C-reactive protein (mg/L)	62,340 (17,505–1,77,600)	–
Length of hospitalization (days)	22 (18–27)	–
CHARLSON index	3 (1–4)	–
ICU hospitalization	–	9 (15%)

*SD, Standard deviation; IQR, Interquartile range; ICU, Intensive care unit*.

### Oral Bacteriotherapy (T0–T1)

Among all patients recruited (*n* = 58), 24 (41.4%) received oral bacteriotherapy during the hospitalization period [median: 23 days (IQR: 19–38 days)] while 34 (58.6%) were treated with only pharmacological treatment [median: 23 days (IQR: 19–38 days)]. Specifically, 70.8% (17/24) of the OB+ patients had received supplemental oxygen therapy, 87.5% (21/24) hydroxychloroquine, 25% (6/24) azithromycin, 50% (12/24) an anti-IL-6 agent (Tocilizumab), and 29.2% (7/24) antiviral therapy. Amongst the OB– group, 85.3% (29/34) of the individuals had received supplemental oxygen therapy, 88.2% (30/34) hydroxychloroquine, 35.3% (12/34) azithromycin, 47% (16/34) an anti-IL-6 agent (Tocilizumab), and 29.4% (10/34) antiviral therapy. Therefore, no statistically significant differences were determined between the OB+ and OB– groups with respect to all clinical characteristics and therapeutic regimens. The characteristics of the two groups are shown in Table 2.

**Table 2:** Demographic and clinical characteristic of OB– and OB+ SARS-CoV-2-infected patients.

**Parameters**	**OB– (*****n*** **=** **34)**		**OB+** **(*****n*** **=** **24)**		***p*-value**
	**Median (IQR 25–75%)**	**Number (%)**	**Median (IQR 25–75%)**	**Number (%)**	
Gender. Male/Female	–	23 (68)/11 (32)	–	14 (58)/10 (42)	0.86–0.66
Age	62 (52–63)		64 (56–69)	–	0.47
White blood cells (mmc)	5,870 (4,390–7,105)	–	5,300 (3,615–7042.5)	–	0.56
Lymphocytes (mmc)	805 (642.5–1137.5)	–	810 (620–1,220)	–	0.86
Lymphocytes (%)	14.6 (9.8–20.1)	–	17.4 (10.8–25.9)	–	0.84
Glucose (mg/dL)	106 (89.75–120.3)		99.5 (85–142.8)		0.92
C-reactive protein (mg/L)	80,480 (24,545–2,02,000)	–	57,120 (14,760–1,29,815)	–	0.23
Length of hospitalization (days)	20.5 (18–26)	–	22.5 (19.3–37.5)	–	0.9
CHARLSON index	2 (1–4)	–	3 (1–5)	–	0.35
ICU hospitalization	–	7 (21)	–	2 (8.3)	0.19

Considering that fatigue has been recently described as one of the listed symptoms of post-COVID-19 (1, 22, 23), at 6 months (T6) after hospital discharge all SARS-CoV-2-infected patients underwent the FAS questionnaire. Out of the 58 patients, 41 (70.7%) reported fatigue [FAS median (IQR) 32(26–36)], while 17 (29.3%) individuals displayed a FAS score below 22 [FAS median (IQR) 20(17–20)] (fatigued FAS vs. not fatigued FAS: *p* < 0.01). At hospital discharge, no significant differences in demographic and clinical variables were determined between subjects found fatigued and not fatigued at T6 (Table 3).

**Table 3:** Demographic and clinical characteristic of fatigued and not fatigued SARS-CoV-2-infected patients.

**Parameters**	**Not fatigued (No. 17)**		**Fatigued (No. 41)**		***p*-value**
	**Median (IQR 25–75%)**	**Number (%)**	**Median (IQR 25–75%)**	**Number (%)**	
Gender, Male/Female	–	10 (59)/7 (41)	–	27 (66)/14 (34)	0.629
Age	62 (51–64)	–	66 (59–70)	–	–
White blood cells (mmc)	5,230 (3,980–6,800)	–	5,900 (4,290–7,120)	–	0.490
Lymphocytes (mmc)	800 (690–1,200)	–	810 (600–1,200)	–	0.875
Lymphocytes (%)	17.3 (13.4–25.9)	–	14.6 (9.6–21.1)	–	0.678
Glucose (mg/dL)	101 (85–126)	–	110 (89–129)	–	0.480
C-reactive protein (mg/L)	22,080 (14,040–1,22,520)	–	68,040 (21,360–1,77,960)	–	0.555
Length of hospitalization (days)	25 (20–43)	–	21 (18–26)	–	**0.030**
CHARLSON index	2 (1–4)	–	3 (1–4)	–	0.095
ICU hospitalization	–	1 (6)	–	8 (20)	0.111

*SD, Standard deviation; IQR, Interquartile range; ICU, Intensive care unit. The bold values indicate statistic significant values*.

A significantly higher proportion of subjects positive for fatigue has been observed in the OB– group compared to those additionally treated with SLAB51 (OB– vs. OB+, 91% (31/34) vs. 41.7% (10/24); *p* < 0.01). The fatigued patients who had received SLAB51 reported significantly lower FAS scores than those not treated with the probiotic formula [median (IQR), OB+, 24 (22.5–26) vs. OB–, 34 (31.5–38); *p* = 0.02]. Interestingly, the proportion of subjects presenting extreme fatigue was significantly higher among the OB– group than in the OB+ one [OB– vs. OB+, 29.4 (10/34) vs. 4.2 (1/24); *p* = 0.047].

### Metabolomic Profile of SARS-CoV-2 Infected Patients

Since CFS has been previously associated to altered metabolic signature (27), we performed metabolomic profiling of the serum collected from our patients at T0 and T1 to evaluate modification in the concentration of key metabolites.

**Figure 1:**
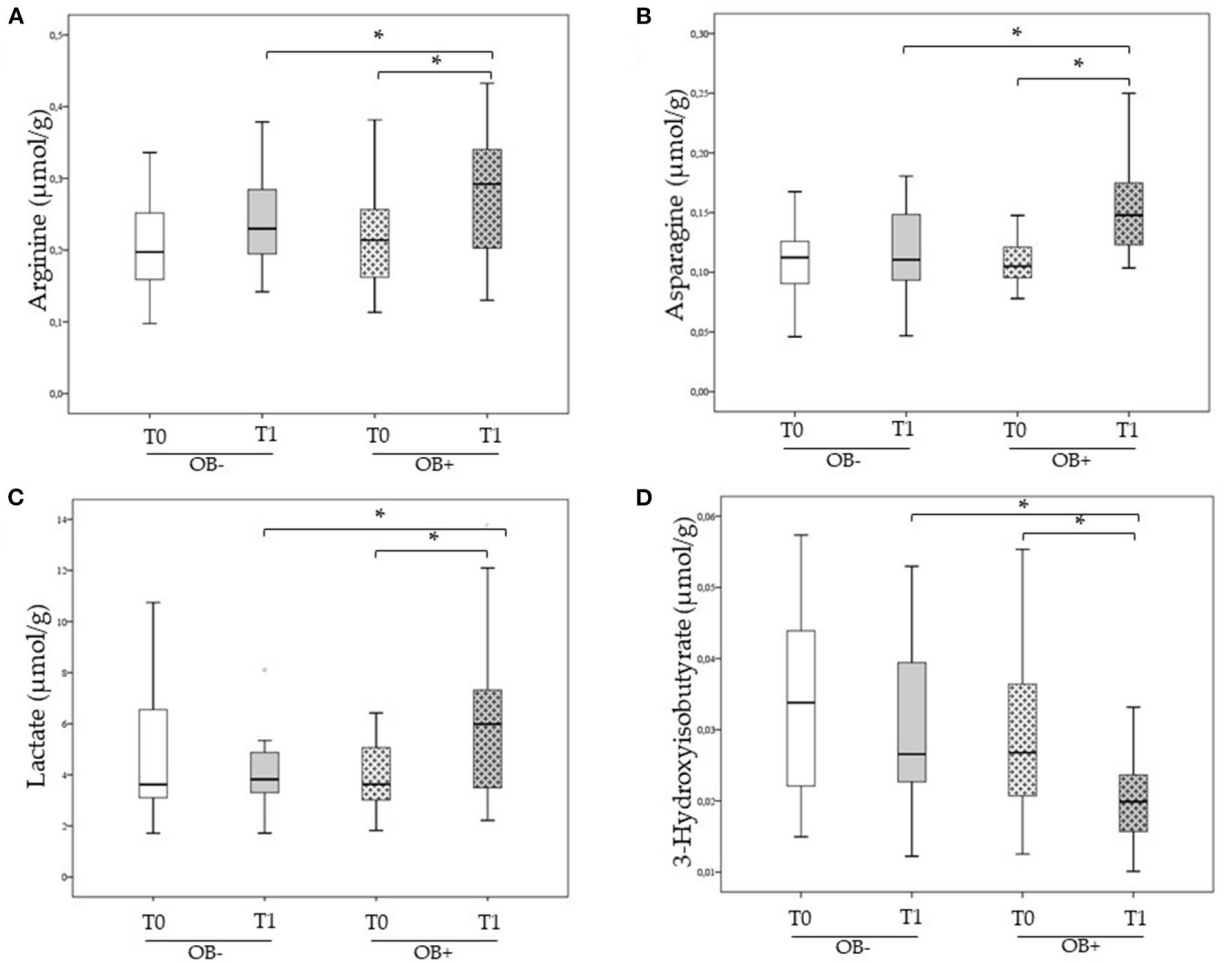
**(A–D)** Serum Arginine, Asparagine, Lactate, and 3-Hydroxyisobutirate concentration in SARS-CoV-2 infected patients receiving and not receiving oral bacteriotherapy treatment at T0 and T1. **(A)** Comparison of Arginine serum concentration at T0 and T1 between SARS-CoV-2 infected patients treated or not treated with OB. **(B)** Comparison of Asparagine serum concentration at T0 and T1 between SARS-CoV-2 infected patients treated or not treated with OB. **(C)** Comparison of Lactate serum concentration at T0 and T1 between SARS-CoV-2 infected patients treated or not treated with OB. **(D)** Comparison of 3-Hydroxyisobutirate serum concentration at T0 and T1 between SARS-CoV-2 infected patients treated or not treated with OB. Data were analyzed using the Mann–Whitney *U*-test and the Wilcoxon signed-rank test for paired samples. *Statistically significant.

Before treatment, the OB– and the OB+ groups were comparable with respect to all serum metabolites considered. Significantly increases in the levels of Arginine, Asparagine, and Lactate opposite to a significantly decrease in the levels of 3-Hydroxyisobutirate have been determined in SARS-CoV-2-infected patients after the SLAB51 treatment (Figure 1). Conversely, no significant modification in the concentration of the same serum metabolites were observed for the OB– group (Figure 1).

### Sub Analysis of Patients Enrolled Not Admitted to Intensive Care Unit

In order to minimize the possible biases between the two groups, we also evaluated our data after removing the patients hospitalized in ICU from both groups (OB– and OB+). Obtained results were as follows: the group administered with probiotics was characterized by a significantly lower prevalence of fatigued subjects with respect to the one not taking oral bacteriotherapy [OB– vs. OB+; 24(70.58%) vs. 9(37.5%), *p* = 0.0003]. The group administered with probiotics was characterized by significantly lower FAS score values than the one not taking oral bacteriotherapy [OB– vs. OB+; median (IQR), 33(31–34.5) vs. 24 (26–22), *p* = 0.007]. For what concerns the metabolic variables no changes has been observed respect to what reported in the manuscript except for Arginine for which no significant differences has been determined between the OB– and the OB+ one at T1 (see Supplementary Figure 1).

### *Post-hoc* Power Analysis

Finally, we performed a *post-hoc* power analysis relative to all endpoint we reported in the manuscript as significantly different. The results of our analysis, reported as Supplementary Table 1, show that for almost considered variables the power of the used statistical methods is more than 50% with most of the major endpoints showing >90% retained power.

## Discussion

An accepted definition of post-COVID-19 fatigue is a “decrease in physical and/or mental performance that results from changes in central, psychological, and/or peripheral factors due to the COVID-19 disease” (28). The prevalence of fatigue has been reported in up to 50% of post-COVID-19 patients (6).

Without evidence of organ damage, these individuals feel tired and unwilling to perform current tasks even after many months from the clearing of the infection. To date, there is no specific tool available to assess fatigue in COVID-19 subjects. Therefore, we used the Fatigue Assessment Scale (FAS), which is convenient for patients to complete and is not time-consuming. Physicians have used FAS for monitoring patients with sarcoidosis and idiopathic pulmonary fibrosis (19–23). We have evaluated the patients 6 months from discharge.

All enrolled subjects were acutely and severely ill at admission to the ER (Emergency Room) (Table 1). As per our internal protocol (11) 34 (58.6%) patients had standard treatment, and 24 (41.4%) also supplementation with the high potency probiotic blend SLAB51. Patients remained hospitalized until clinical resolution (SO_2_ ≥94%) or testing negative for COVID-19. At discharge, all the patients had similar non-detectable blood levels of serotonin, dopamine, or acetylcholine (Figure 1). They had been hospitalized and isolated for a long time, and therefore and therefore they appeared psychologically relieved due to discharge.

Six months after release from the hospital, no patient showed abnormal lung function tests or SO_2_ below 94%. We could not assess the role of family environment, economic conditions, and other feelings during their 6 months stay at home with their families. Still, none of the patients referred to the use of antidepressants, sedatives, or sleeping pills before the FAS test. A significantly lower proportion of subjects experimenting CFS has been determined in the group additionally administered with SLAB51 with respect to that taking only standard pharmacological therapy. Notably, the group administered with SLAB51 presented also a significantly lower percentages of subjects with extreme fatigue, compared to the group not taking probiotics.

Patients with symptoms as fatigue, “brain fog,” pain, breathlessness, and orthostatic intolerance (29) are more and more frequent and suggest chronic involvement of the central nervous system (CNS) (30). New findings show that detectable levels of the virus in COVID-19 in the brains are low and do not correlate with histopathological changes (31). The observed microglial activation, microglial nodules, and neuronal phagocytosis result from systemic inflammation, probably in synergy with hypoxia and ischemia (32, 33). As detected by 18F-fluorodeoxyglucose positron emission tomography (FDG-PET), which measures brain glucose metabolism, the hypometabolism of the frontal lobe may be involved in fatigue in patients with COVID-19 (34). Undeniably, in subjects with post-COVID-19 fatigue, the metabolic homeostasis is disturbed (35, 36).

The metabolomic profile of the two groups was similar at hospital admission. At T1, increased serum levels of arginine, asparagine, lactate, and decreased levels of 3-Hydroxyisobutyrate were found in OB+ treated subjects (*n* = 24). The same parameters did not change among OB– patients at T1.

Six months later, when the FAS test was administered, the group additionally administered with SLAB51 presented a significantly lower prevalence of chronic fatigue as compared to patients treated with only the pharmacological therapy. At T1, the subjects treated with SLAB51 had significantly higher levels of arginine as compared to baseline and to the OB– group at T1. Although this observation needs to be confirmed by investigating larger number of samples, our results strongly suggest that the arginine levels/variations might constitute an early predictive marker of chronic fatigue when COVID-19 patients are discharged from the hospital. We hypothesize that the higher levels of arginine observed in patients treated with SLAB51 at discharge may underlie the better physical and psychiatric conditions observed after 6 months, compared to subjects treated only with standard therapy.

In this context, arginine is essential in the regulation of growth hormone, glucagon, insulin, and for the synthesis of DNA, RNA, polyamines, and creatine, a molecule produced in the liver to regenerate ATP following muscle contraction (37, 38). Low concentrations of arginine have been consistently associated with fatigue in its chronic and reversible forms. Mizuno et al. found that plasma concentration of arginine, together with other branched-chain amino acids, was low in healthy subjects after mentally fatiguing activities (39). In addition, Camic et al. found that the administration of arginine was able to delay the onset of neuromuscular fatigue (40). According to Chen et al. this could be related to the arginine ability to remove the NH3 in excess by increasing nitric oxide biosynthesis or urea cycle (41).

It is also interesting to point out the pattern of 3-Hydroxyisobutyrate that decreases in subjects treated with SLAB51 (OB+) but remains unmodified in those not treated with oral bacteriotherapy (OB–). 3-Hydroxyisobutyrate in conditions of energy deficiency is a source of nourishment for the heart, brain, and muscles. It has been recently published that ketone bodies are unbalanced in subjects with COVID-19 and that their persistence at high levels in the blood was associated with a poor prognosis (42–44). In the OB– group, the unmodified levels of 3-Hydroxyisobutyrate suggest steady state compensatory mechanisms for altered glucose utilization or insulin resistance. The treatment with SLAB51 might have lowered 3-Hydroxyisobutyrate in the OB+ group by modifying arginine and asparagine levels that regulate glucagon and insulin and consequently improved glucose utilization and energy metabolism. Asparagine in the body is a critical support in energy production and is essential for nervous tissue, particularly brain development and function (45–47). In addition, it can increase the synthesis of glucagon, the hormone that antagonizes insulin, which causes an increase in blood glucose. Low levels of asparagine may indicate poor metabolism or synthesis of aspartic acid, resulting in the inability to synthesize and excrete urea appropriately. The inability to excrete urea can result in the formation of toxic nitrogen-containing metabolites, which can cause a variety of symptoms, such as depression, irritability, headaches, confusion, or, in more extreme cases, psychosis. Decreased arginine and asparagine levels are present in the plasma during acute pulmonary inflammatory exacerbations (48).

The lactate increase observed only in OB+ subjects should be framed in the context of energy metabolism utilization. We do not think that the difference observed at T1 results from a different muscle activity in our patients. The OB+ and OB– subjects had been bedridden for weeks and discharged without any motor rehabilitation. Therefore, in our opinion, the observed variation depends on the modification of the intestinal flora induced by SLAB51. It is well-known that appropriate intestinal fermentation is associated with improved physical and athletic performance (49). An increase in lactate levels in OB+ subjects could be an additional energy source for the brain, as suggested by its use as a neuroprotectant in brain trauma (50).

The observational nature of the study, as well as the lack of appropriate control, randomization, and blinding should be assumed as major limitations for this study; taking into account all those aspects, the authors consider the results suggestive for generating hypothesis that will need further confirmatory studies, but currently incomplete to draw firm conclusions.

## Conclusion

In conclusion, a growing body of evidence suggests that dysbiosis and modification of metabolic processes due to the imbalance of the intestinal flora, observed in patients infected by SARS-CoV-2, may play a key role in determining the severity of the COVID-19, including neuropsychological, psychiatric and long-term disorders (51–55). Given the significant impact of SARS-CoV-2 on host's microbiota and the current lack of effective therapeutic options, a possible role of oral bacteriotherapy has been hypothesized, as complementary therapeutic strategy to fight the pathophysiological changes due to SARS-CoV-2 and the COVID-19 related damages (11–15, 56–62).

We believe that, in addition to what has been previously reported regarding survival and the risk of being intubated (11), the administration of SLAB51 during hospitalization may affect the subject's performance months later. Even considering all the limitations of the present study, some of the evidence can be underlined: (a) subjects infected by the virus, if treated with SLAB51, have a lower incidence of chronic fatigue; (b) the administration of bacteriotherapy induces metabolic changes for a better utilization of glucose and energy pathways. Nevertheless, these findings need to be confirmed by more in-depth analysis and replicated in further studies.

## Data Availability Statement

The raw data supporting the conclusions of this article will be made available by the authors, without undue reservation.

## Ethics Statement

The studies involving human participants were reviewed and approved by Ethics Committee of Policlinico Umberto I—Sapienza University (approval number: Rif. 109/2020). The patients/participants provided their written informed consent to participate in this study.

## Author Contributions

Gd'E and GC: conceptualization and validation. LS, LL, GI, CP, and GC: methodology. LS, GI, CP, PV, AL, and CB: formal analysis. LS, LL, GI, CP, LC, LT, and AK: investigation. LS, LL, GI, CP, LC, and MM: data curation. LS, GI, CP, PV, MM, Gd'E, and GC: writing—original draft preparation. LS and Gd'E: writing—review and editing. CM, Gd'E, and GC: visualization. Gd'E: supervision, project administration, and funding acquisition. All authors have read and agreed to the published version of the manuscript.

## Funding

This work was supported in part by grants of Sapienza University of Rome (Progetti di Ateneo 2018 N.801/2019).

## Conflict of Interest

The authors declare that the research was conducted in the absence of any commercial or financial relationships that could be construed as a potential conflict of interest.

## Publisher's Note

All claims expressed in this article are solely those of the authors and do not necessarily represent those of their affiliated organizations, or those of the publisher, the editors and the reviewers. Any product that may be evaluated in this article, or claim that may be made by its manufacturer, is not guaranteed or endorsed by the publisher.
