# Supplementary material for: Oral Bacteriotherapy Reduces the Occurrence of Chronic Fatigue in COVID-19 Patients
Source: Front Nutr. 2022 Jan 12;8:756177. doi: 10.3389/fnut.2021.756177 (PMC8790565; doi:10.3389/fnut.2021.756177)

**Supplementary table S1:** *Post hoc* power analysis relative to all endpoint we reported in the manuscript as significantly different.

| Variable | OB- | | OB+ | | Used test | Statistical power (%) |
| --- | --- | --- | --- | --- | --- | --- |
|  | Mean±SD | No.(%) | Mean±SD | No.(%) |  |  |
| Subjects positive to fatigue |  | 31(91%) |  | 10(41,7%) | Chi square test | 98,9 |
| FAS fatigued subjects | 33,74±4,94 |  | 26,2±5,75 |  | Mann-Whitney U test | 96 |
| Lactate T1 | 4,23±1,85 |  | 6,16±3,04 |  | Mann-Whitney U test | 79,1 |
| Asparagine T1 | 0,12±0,04 |  | 0,15±0,04 |  | Mann-Whitney U test | 86,5 |
| Arginine T1 | 0,24±0,06 |  | 0,28±0,08 |  | Mann-Whitney U test | 52,3 |
| 3-Hydroxyisobutyrate T1 | 0,03±0,02 |  | 0,02±0,01 |  | Mann-Whitney U test | 91,2 |
| Lactate T0vsT1 |  |  | 4,23±1,75 vs 6,16±3,04 |  | Wilcoxon signed rank test | 66,3 |
| Asparagine T0vsT1 |  |  | 0,11±0,03 vs 0,15±0,04 |  | Wilcoxon signed rank test | 74,5 |
| Arginine T0vsT1 |  |  | 0,22±0,07 vs 0,28±0,08 |  | Wilcoxon signed rank test | 78,1 |
| 3-Hydroxyisobutyrate T0vsT1 |  |  | 0,03±0,01 vs 0,02±0,01 |  | Wilcoxon signed rank test | 94 |

Post hoc statistical power of applied test with respect to clinical and metabolic variables found reported as significant in the manuscript. Analyses have been performed by using the power analysis module included in XLstat v. 2019.2.2 (Addinsoft, USA).

**Supplementary figure S1:** Serum Arginine, Asparagine, Lactate and 3-Hydroxyisobutirate concentration in SARS-CoV-2 infected patients receiving and not receiving oral bacteriotherapy treatment at T0 and T1. Sub analysis in patients not admitted to intensive care unit


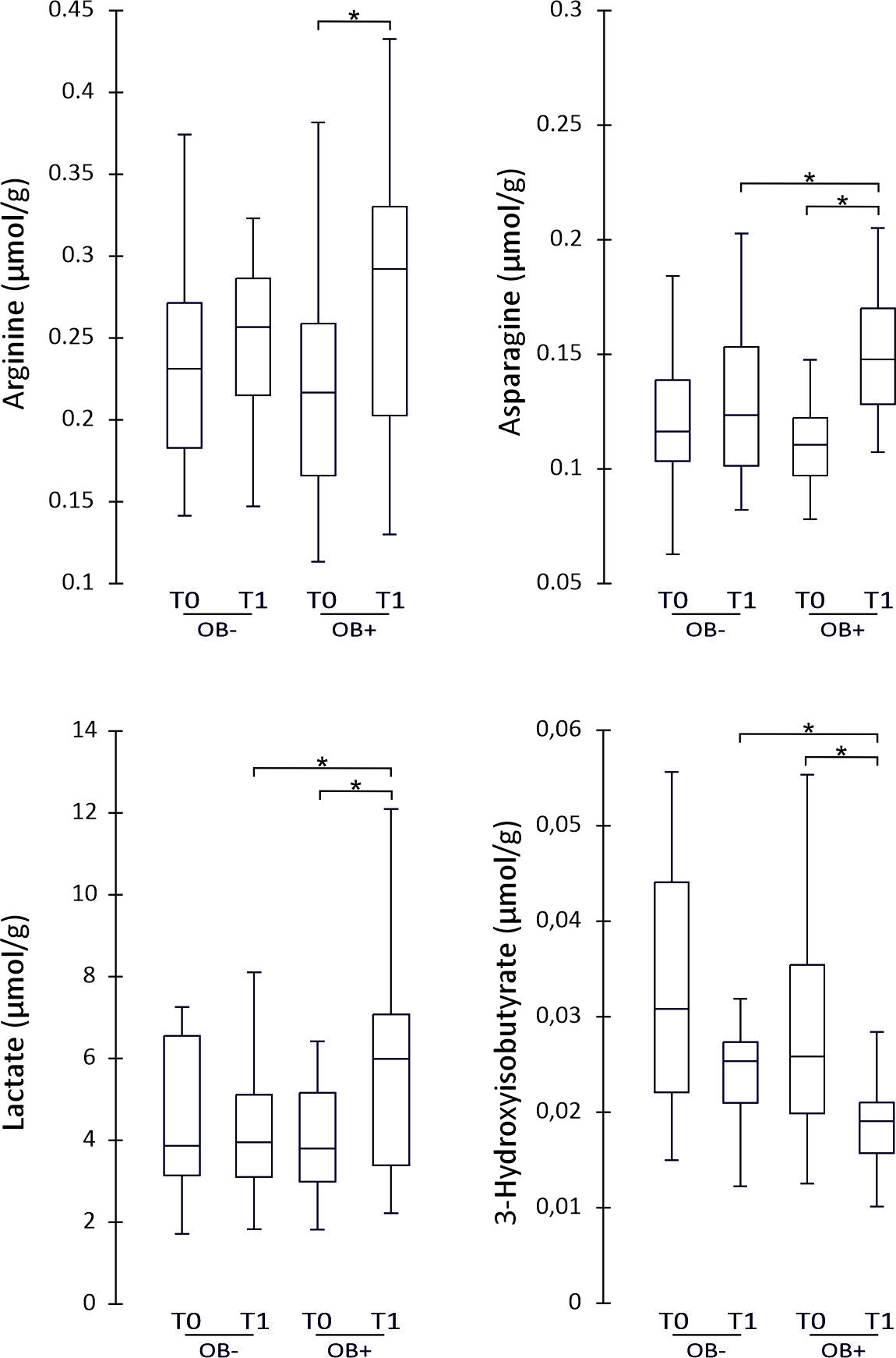

Supplement: Supplementary file 1 [file Data_Sheet_1.docx]
